# Enhancing Extraction of Drug-Drug Interaction from Literature Using Neutral Candidates, Negation, and Clause Dependency

**DOI:** 10.1371/journal.pone.0163480

**Published:** 2016-10-03

**Authors:** Behrouz Bokharaeian, Alberto Diaz, Hamidreza Chitsaz

**Affiliations:** 1 NIL Group, Complutense University of Madrid, Ciudad Universitaria, Calle Profesor José García Santesmases, 28040 Madrid, Spain; 2 Department of Computer Science, Colorado State University, Fort Collins, Colorado, United States of America; University of Lisbon, PORTUGAL

## Abstract

**Motivation:**

Supervised biomedical relation extraction plays an important role in biomedical natural language processing, endeavoring to obtain the relations between biomedical entities. Drug-drug interactions, which are investigated in the present paper, are notably among the critical biomedical relations. Thus far many methods have been developed with the aim of extracting DDI relations. However, unfortunately there has been a scarcity of comprehensive studies on the effects of negation, complex sentences, clause dependency, and neutral candidates in the course of DDI extraction from biomedical articles.

**Results:**

Our study proposes clause dependency features and a number of features for identifying neutral candidates as well as negation cues and scopes. Furthermore, our experiments indicate that the proposed features significantly improve the performance of the relation extraction task combined with other kernel methods. We characterize the contribution of each category of features and finally conclude that neutral candidate features have the most prominent role among all of the three categories.

## Introduction

Extracting biomedical relations from texts is a relatively new, but rapidly growing research field in natural language processing. Owing to the increasing number of biomedical research publications and the key role of databases of biomedical relations in biological and medical research, extracting biomedical relations from scientific articles and text resources is of utmost importance. Drug-drug interaction (DDI) is, in particular, a widespread concern in medicine, and thus, extracting this kind of interaction automatically from texts is of high demand in BioNLP. Drug-drug interaction usually occurs when one drug alters the activity level of another drug. According to the reports prepared by the Food and Drug Administration (the FDA) and other acknowledged studies [1], over 2 million life-threatening DDIs occur in the United States every year. Many academic researchers and pharmaceutical companies have developed relational and structural databases, where DDIs are recorded. Nevertheless, most up-to-date and valuable information is still found only in unstructured research text documents, including scientific publications and technical reports.

In this paper, we first introduce the basics of three complementary, linguistically driven feature sets of (i) negation, (ii) clause dependency, and (iii) neutral candidates. The ultimate aim of this research is to enhance the performance of DDI extraction task by considering and employing the above-mentioned three operations and feature sets.

First, it is essential to detect negative assertions in most biomedical text-mining tasks, where the overall purpose is to derive factual knowledge from textual data. According to Loos et al. [2], negation is a morphosyntactic operation in which a lexical item denies or inverts the meaning of another lexical item or construction. Likewise, a negator is a lexical item that expresses negation. Negation is commonly utilized in biomedical articles and is an important origin of low precision in automated information retrieval systems [3]. Generally, two negation detection methods have been developed and employed for annotating the applied corpora: a linguistic-based approach and an event-oriented approach. Two of the known negation annotated corpora are the linguistically focused, scope-based *BioScope* and the event-oriented *Genia* [4].

Second, identifying the role of clause dependency in complex sentences in DDI detection is another linguistically driven subject which is investigated in this research. According to Harris and Rowan [5], a dependent clause is a group of words with a subject and a verb that do not express a complete thought, cannot stand alone, and usually extend the main clause. An independent clause, or main clause, is one that can stand alone as a sentence and express a complete thought. Consequently, a complex sentence has one independent clause and at least one dependent clause. Moreover, a clause connector is a word that joins clauses in order to form complex sentences. Coordinators, conjunctive adverbs, and subordinators are three types of connectors.

Miwa et al. [6] have considered clauses in relation extraction task. They have reported some improvements regarding different types of simplification and clause selection rules which they have applied. By contrast, in this research we extract new features based on the text or subtree features in a kernel-based relation extraction method. Our features detect the existence token or subtree in a dependent or independent clause as well as the type of the clause itself via checking several clause connectors.

Finally, we study the role of neutral DDI candidates in the relation extraction. Most of the current relation extraction problems and the produced corpora are based on binary relations; they decide a binary relation between two entities. Similarly, in the DrugDDI corpus [7], the implemented systems must predict whether or not an interaction between the two drugs has occurred. Although detecting DDI interactions is the main target of the DrugDDI corpus, there is a difference between a negative interaction candidate having been stated by the authors (distinguished candidate) and that which has not (neutral candidate). Both of these candidates are considered negative in DrugDDI corpus. In other words, the neutral interaction candidate is a co-mention of two drugs with no remarks by the author in the sentence or the discussed clause, while the distinguished interaction candidate is exactly the opposite (with remarks by the author). In point of fact, neutral candidates are a particular subclass of non-positive candidates whose lack of interaction cannot be exactly determined by the confident level above zero. For instance, consider the following sentence:

- Studies in healthy volunteers have shown that acarbose has no effect on either the pharmacokinetics or pharmacodynamics of digoxin, nifedipine, propranolol, or ranitidine.

There is no remark by the author about the interaction between *propranolol* and *ranitidine*. Therefore, we define this candidate of drug-drug interaction as a neutral candidate.

One among the few studies on detection of neutral candidates has been conducted by [8], introducing two iteration-based systems of DIPRE and Snowball that take into account the confidence level of the relation. In both systems, when the confidence level is zero, there is a neutral candidate. Moreover, Frunza and Inkpen have carried out another similar research which considers neutral candidates [9]. They categorize and extract the semantic relationships between disease and treatments from biomedical sentences. However, no significant improvement has been reported through using neutral class in the work.

In the present study, we characterize the role and the potential importance of the three above- mentioned categories of features in DDI extraction. We employ the combinations of the extracted features along with the existing well-established kernel methods. For instance, the status of a neutral DDI candidate is not inverted when negation is used, whereas a non-neutral candidate is inverted. In addition, when a negator is added, the overall status of a DDI candidate may or may not be reversed, depending on the type of the clause connector that contains DDI candidate and negator. This issue will be expounded in the methods section.

The rest of the paper is structured as follows. The following section provides the background in some of the kernel-based relation extraction methods, beneficial NLP subtasks, and some of the related data sources. In section 3, we present our approach and the feature extraction process, and section 4 is devoted to presenting the results obtained. The final section concludes the paper and gives some suggestions for future research.

## Background

The majority of previous works on biomedical relation extraction, including the DDI detection, have been carried out on the basis of supervised binary relations extraction [8]. In this paper, we summarize kernel-based relation extraction methods as well as some NLP preprocessing enhancements and the related corpora.

### 2.1 Kernel-based methods

Sequence kernels [10], Tree kernels such as parse tree based [11], and Graph kernels such as graph parsing [12] are among the most important kernel-based methods [13]. Two more recent approaches have been proposed by [14] and [15], being ranked first and second in DrugDDI challenge (2013), respectively. Chowdhury and Lavelli [14] proposed a hybrid kernel through linear combination of a feature-based kernel, a Shallow Linguistic (SL) kernel, and a Path-Enclosed Tree (PET) kernel. Through defining a multiplicative constant, they assigned more (or less) weight to the information obtained by tree structures. Another recent work has been accomplished by [16] who employed a feature-based linear kernel that contains five categories of features, including word pair and dependency graph features. In addition to the previous methods, a number of research have improved the performance of the task through ensemble approaches. For example, Thomas and his colleagues [15] proposed a two-step approach in which the relation candidates are initially extracted, using the ensembles of up to five different classifiers and then are relabeled to one of the four used categories in the task. The other work which has been suggested by He and her colleagues [17] applies a stacked generalization approach to learn the weights which have been exploited to combine graph and tree kernels.

### 2.2 NLP enhancements

Several related NLP enhancements have improved the performance of the relation extraction algorithms. They are often employed as a preprocessing step which is a pivotal stage in enhancing the overall performance and results. In particular, we summarize the studies on negation, sentence, and clause simplification.

Faisal et al. [18] took negation into account in the relation extraction task. They developed a list of features, such as the nearest verb to the candidate entities in the parse tree and few negation cues, which are fed into an SVM classifier. They reported some improvements, but did not specify how much the negation identification step enhanced the performance.

Another NLP enhancement in the relation extraction is sentence and clause simplification to overcome the complexity of the sentences. Text simplification modifies, enhances, classifies, or otherwise processes an existing text in such a way that the grammar and the structure of the prose are simplified to a great extent, while the original meaning and information remain the same [19]. ISIMP is a system that simplifies the text so that its mining tools, including the relation extraction tasks, can be improved [20]. In the same direction, Segura and her colleagues proposed techniques to simplify complex sentences by splitting the clauses [21]. They applied some rules and patterns to split the clauses and then utilized some simplification rules to generate new simple sentences. However, according to their conclusion, difficulty of resolving nested clauses is the major source of errors. There are other NLP subtasks enhancements that can be employed in the relation extraction task, although they were not applied in our work. To name a few, Velldal et al. [22] proposed speculation detection, and Lappin [23] utilized anaphora resolution.

### 2.3 Related corpora

#### DrugDDI corpora

Drug-Drug Interaction corpus was primarily developed by Segura and Mart [7], with 579 XML files describing DDIs which were collected randomly from the DrugBank database [24]. The first DDI Extraction competition was held in 2011 with the aim of encouraging researchers to explore new methods for extracting drug-drug interactions [7]. A second competition was held in 2013 as part of SemEval-2013 (International Workshop on Semantic Evaluation). Furthermore, a new corpus was developed which included the corpus used in 2011 (DDI-DrugBank, 2011) as well as some MEDLINE abstracts. The teams participating in this venue had developed solutions based on supervised and sentence-level relation extraction methods, and the best F-measure achieved was 75% [25].

#### Corpora annotated with negation

As mentioned earlier, thus far two negation detection methods have been developed and employed for annotating the corpora utilized: a linguistic-based approach and an event-oriented approach. Linguistically-focused BioScope and the event-oriented Genia [4] are two of the known negation annotated corpora.

In BioScope, the scopes aim to recognize the negation position of the key event in the sentence and with each argument of these key events was located under the negation scope as well [26]. In contrast, Genia deals with the modality of events within the events independently. In the Genia event, biological concepts (relations and events) are annotated for negation, but no linguistic cues are annotated for them. In point of fact, the main objective of the BioScope corpus is to investigate this language phenomenon in a general, task-independent, and linguistically-oriented manner. Additionally, in the BioScope, in-sentence negation scope and cues can be recognized automatically [4].

#### NegDDI-DrugBank corpus

**Fig 1:**
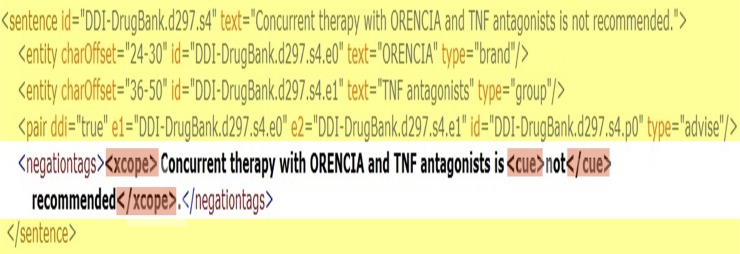
The extended unified XML format of a sentence with negation cue in NegDDI-DrugBank corpus.

Konstantinova et al. developed two corpora [27] and Morante and Blanco [28] adapted Bioscope’s guidelines. These adaptations in addition to the previously mentioned advantages of the bioscope annotations prove them to be a valuable resource. Consequently, we produced NegDDI-DrugBank corpus based on the Bioscope’s guidelines. For this purpose, all sentences of DrugDDI (2011) and DrugBank part of the DrugDDI (2013) were utilized and automatically annotated. Bokharaeian et al. [29] explained the annotation process and presented a detailed analysis of the number of distinct negation cues in the NegDDI-DrugBank corpus. The extended corpus is available for public use [30]. A sample of the extended negation annotation can be seen in Fig 1. The negation scope and the cue xml tags are highlighted in the extended part which is transparent in this figure.

## Methods

In this section, the feature extraction phase as well as the proposed method for the DDI prediction will be presented. Our features, presented in Table 1, are categorized into three major categories based on the linguistic definition of negation, the position of the drugs discussed in the sentence, and the linguistic-based confident level of an interaction: (i) negation scope and cue-related features, (ii) clause dependency features, and (iii) neutral candidate’s features. In all of the presented tables, “NEG” has been used as the abbreviation for the negation scope and cue feature set, and “CLA” and “NEUT” stand for the clause dependency feature set and the neutral candidate feature set, respectively. Moreover, it is worth mentioning that all of the sample sentences in this paper have been obtained from the DrugDDI corpus [25].

**Table 1:** The list of the extracted features used in the system.

Feature category	Feature name	Type	Definition
**Negation Scope and Cue**	BothInsideNegSc	Boolean	is set as true when both drugs are inside the negation scope
	BothRightNegSc	Boolean	is set as true when both drugs are on the right side of the negation scope
	BothLeftSNegSc	Boolean	is set as true when both drugs are on the left side of the negation scope
	OneLeftOneInsideNegSc	Boolean	is set as true when one drug is on the left side of the negation scope, and the otheron the inside
	OneRightOneInsideNegSc	Boolean	is set as true when one drug is on the right side of the negation scope, and theother on the inside
	OneLeftOneRightSc	Boolean	is set as true when one drug is on the right side of the negation scope, and theother on the left
	NegationCue	String	Negation cue
**Clause Dependency Detection**	AlthoughIS	Boolean	set as true when the sentence has *although* token
	WhileIS	Boolean	set as true when the sentence has *while* token
	WhenIS	Boolean	set as true when the sentence has *when* token
	BeforeIS	Boolean	set as true when the sentence has *before* token
	NowthatIS	Boolean	set as true when the sentence has *now that* token
	AssoonasIS	Boolean	set as true when the sentence has *as soon as* token
	AslongasIS	Boolean	set as true when the sentence has *as long as* token
	AnywhereIS	Boolean	set as true when the sentence has *anywhere* token
	UntilIS	Boolean	set as true when the sentence has *until* token
	OnceIS	Boolean	set as true when the sentence has *once* token
	TillIS	Boolean	set as true when the sentence has *till* token
	BecauseIS	Boolean	set as true when the sentence has *because* token
	ThoughIS	Boolean	set as true when the sentence has *though* token
	EventhoughIS	Boolean	set as true when the sentence has *even though* token
	SinceIS	Boolean	set as true when the sentence has *since* token
	ButIS	Boolean	set as true when the sentence has *but* token
	UnlessIS	Boolean	set as true when the sentence has *unless* token
	afterIS	Boolean	set as true when the sentence has *after* token
	whereasIS	Boolean	set as true when the sentence has *where* token
	asthoughIS	Boolean	set as true when the sentence has *as though* token
	sothatIS	Boolean	set as true when the sentence has *so that* token
	inorderthatIS	Boolean	set as true when the sentence has *in order to* token
	everywhereIS	Boolean	set as true when the sentence has *everywhere* token
	evenifIS	Boolean	set as true when the sentence has *even if* token
	RatherthanIS	Boolean	set as true when the sentence has *rather than* token
	AslongasIS	Boolean	set as true when the sentence has *as long as* token
	OnlyifIS	Boolean	set as true when the sentence has *only if* token
	JustasIS	Boolean	set as true when the sentence has *just as* token
	F-StructuresDependencies	String	Corresponding to every feature F of the original method which contains only tokens or subtrees, if the token or subtree X located in an independent clause, a string X-IDC added to this new feature, otherwise if the token or subtree X located in a dependent clause, a string X-DC added to this new text feature
**Neutral Candidate Detection**	NeutralCandRule1	Boolean	(.)*d1(/|s|()d2(.)
	NeutralCandRule2	Boolean	d2 ||d1.contains(OtherNs(d2)) ||(d2.contains(OtherNs(d1))
	NeutralCandRule3	Boolean	(.)*d1((|s) (N,|e.g.|i.e.|s|DrgNaOth|,|))* d2(.)*
	NeutralCandRule4	Boolean	(.)*d1(s)*,(s|DrgNaOth|,|, and|, other|oral)*d2(.)*
	NeutralCandRule5	Boolean	(.)*(:|such as|e.g.|i.e.)(s|DrgNaOth|,|and|or|and/or)*d1(s|DrgNaOth|,|and)*d2(.)*
	NeutralCandRule6	Boolean	(.)* (been studied)(.)*
	NeutralCandRule7	Boolean	(.)* been investigated (.)* & (.)*(although)(.)*
	NeutralCandRule8	Boolean	(.)* (been established)(.)*
	NeutralCandRule9	Boolean	(.)*(studies)(.)* (performed)(.)*& (.)*(studies)(.)* (conducted)(.)*
	NeutralCandRule10	Boolean	[(.)*][no experience][(.)*]

Additionally, as previously mentioned, DDI Extraction (2013) datasets also include 233 MEDLINE abstracts in addition to the obtained DrugBank texts. This extension was carried out due to dealing with different types of texts and language styles [25]. While, DDI-DrugBank texts focus on the description of drugs and their interactions, the main topic of DDI-MEDLINE texts does not necessarily focus on DDIs. Consequently, in addition to the annotation of the DrugBank part of the corpus, we annotated the MEDLINE part with negation scope and cue. The annotation process was carried out in a similar way to the above-mentioned DrugBank part. The prepared corpus is available at this address (https://figshare.com/s/b657c8ccfa152ed8a426)

### 3.1 Negation scope and cue features

**Fig 2:**
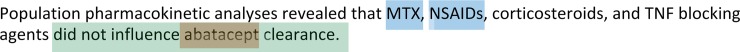
A sample of a negated sentence with some DDI candidates.

In negative sentences, the relative position of the entities compared to the negation scope and cue is an important factor that can be extracted directly from the extended corpus. For instance, consider the negated sentence in Fig 2 [31]. As can be seen in this figure, the scope of negation is highlighted in green.

In the sentence, *MTX* and *NSAIDs*, which have been highlighted in the image, are two drug names that are located outside the negation scope, and consequently, their interaction status is not inverted by negation. However, *abatacept* and *MTX* interaction status is inverted by negation due to the position of *abatacept* located in the negation scope. Regarding the position of drug names inside or outside the negation scope, there are 6 different possibilities used as the six features:

1. BothInsideNegSc: A Boolean feature which is set true when both drugs are inside the negation scope and is set false in all other situations.
2. BothRightNegSc: A Boolean feature which is set true when both drugs are on the left side of the negation scope and is set false in all other situations.
3. BothLeftSNegSc: A Boolean feature which is set true when both drugs are on the right side of the negation scope and is set false in all other situations.
4. OneLeftOneInsideNegSc: A Boolean feature which is set true when one drug is on the left side of the negation scope and the other drug is inside it. The Boolean feature is set false in all other situations.
5. OneRightOneInsideNegSc: A Boolean feature which is set true when one drug is on the right side of the negation scope and the other drug is inside it. The Boolean feature is set false in all other situations.
6. OneLeftOneRightSc: A Boolean feature which is set true when one drug is on the right side of the negation scope and the other drug is on its left side. The Boolean feature is set false in all other situations.

In addition to these six features, the negation cue is utilized as a text feature.

### 3.2 Clause dependency features

**Fig 3:**
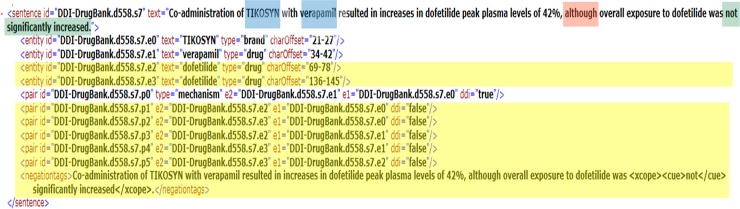
A sample of a negated sentence with a concessive clause.

Previous studies generally indicate that complex and compound sentences, which are very common in the biomedical literature, produce more errors than simple sentences with one clause [21]. Thus, distinguishing between independent and dependent clauses is a critical matter. The analyses demonstrate that more than 27% of the sentences in the test part of NegDDI-DrugBank and 19% of the sentences in the training part of NegDDI-DrugBank have at least one dependent clause. Since a large number of sentences have more than one clause in complex structures, taking clause dependency features into account is important. However, there are different types of dependent clauses that can alter the overall meaning of a sentence in different ways. For instance, *concessive* clause is a clause which begins with “although” or “even though” and expresses an idea that suggests the opposite of the main part of the sentence, like in the sentence shown in Fig 3. The sentence also has one negation cue and scope which has been highlighted in green, and the two drug candidates are highlighted in blue. The clause connector is highlighted in red.

**Fig 4:**
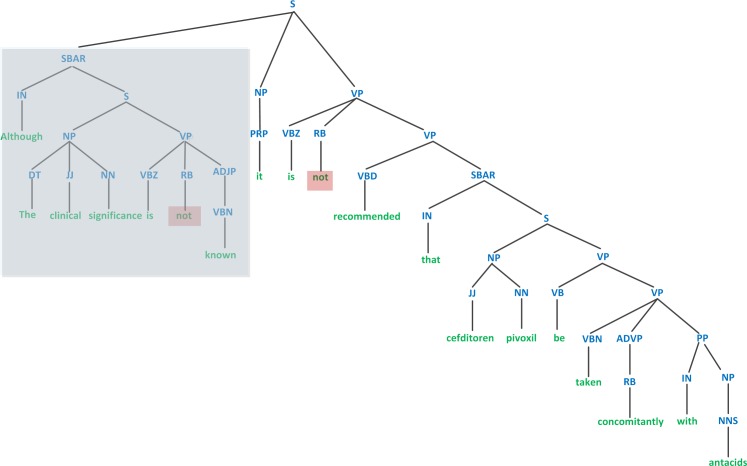
A constituency parse tree of a sentence with a concessive dependent clause highlighted in blue and two negation cues.

The main clause (“*Co-administration of TIKOSYN with verapamil resulted in increases in dofetilide peak plasma levels by 42%*.”) conveys a meaning opposite to that of the dependent clause (“*Overall exposure to dofetilide did not significantly increase*.”). As another example, a graphical view of a parse tree for a complex sentence with a highlighted dependent clause and two highlighted negation cues is presented in Fig 4 [31]. Although it appears that the first clause conveys the same idea as the main clause expresses, the dependent clauses carry less important information than do the main clauses from a linguistic point of view. This point has been neglected in most previous methods, particularly in the sequence kernels.

The next most frequent type of clause in the corpus is adverbial clauses of time that indicate the time of a DDI prevalent in the pharmacological literature. The analysis carried out in the corpus shows that the most frequent adverbial clause connectors are “when”, “while”, and “before”. They collectively constitute approximately half of the total clause connectors.

Considering the different types of dependent clauses, two categories of features were extracted. The first group consists of 28 Boolean features corresponding to 28 clause connectors. The complete list of those connectors as well as their corresponding features is presented in Table 1. The second group of features is based on the substructures (token or subtree) utilized in the applied method, which locates whether the substructure is inside the main clause or not. Three new text features, similar to the features used in the Global context kernel [32], were extracted with IDC prefix for independent clause tokens and DC for dependent clause tokens. Similarly, to improve the subtree kernel, we defined new subtrees. In short, the subtree inside a dependent or independent clause comes with DC or IDC prefix beside the root name, respectively.

### 3.3 Neutral candidate features

**Fig 5:**
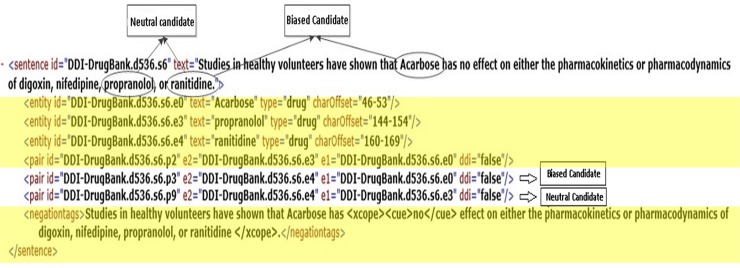
A sample sentence with negation from NegDDI-DrugBank with neutral and distinguished false DDIs.

As it was previously explained, the distinction between distinguished and neutral interaction candidates is critical. A neutral candidate is one with no remark by the author in the sentence, while a distinguished candidate is exactly the opposite (with remarks by the author). In point of fact, neutral candidates are a particular subclass of non-positive candidates that are detectable by meticulously defined features. However, a distinguished candidate can belong to the positive or negative class of DDI’s. In the sentence in Fig 5, the two mentioned interaction candidates are shown. The status of the interaction candidate between each of the discussed drugs at the end of the sentence, i.e. *Propranolol*, *Ranitidine*, etc. is neutral because there are no remarks by the author about their interaction with each other. However, the status of the relation between Acarbose and *Ranitidine*, *Propranolol*, and the other mentioned drugs is distinguished since the author explicitly explains the lack of interaction (“…*Acarbose has no effect on either the*…”).

From the negation action perspective, a negation cue inverts the distinguished candidate, but does not invert the status of a neutral interaction candidate. For instance, in the sentence in Fig 5, the negation has inverted the status of the distinguished candidate *Acarbose* and *Ranitidine* from positive into negative. However, it has not changed the status of the neutral candidates *Propranolol* and *Ranitidine*, and thus, the interaction has remained negative.

In more precise terms, a DDI candidate is called neutral if it has the following two properties:

1. The interaction or lack of interaction between two drugs cannot be extracted from the sentence (or container clause) with confidence level more than zero.
2. The status of the interaction or lack of interaction between two drugs does not change from positive to negative or vice versa if the sentence (or container clause) is negated and drug names are located in the scope of the negation.

**Fig 6:**
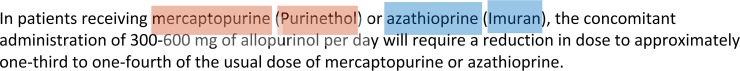
A sample of a sentence having two neutral DDI candidates.

It is worth mentioning that being a neutral candidate can be defined in different linguistic scopes such as a clause, sentence, or a paragraph. In the present paper, a neutral candidate is defined in the scope of the container clause and sentence. Accordingly, 10 Boolean features have been defined concerning linguistically different patterns to detect neutral candidates in the clause and sentences (Table 1). A rule-based system was implemented, using regular expression language beside to some defined functions to extract the features below. In the table, java regular expression patterns have been utilized to mention the rules [33]. In addition to the used patterns, some predefined variables and functions were used in the written rules such as “DrgNaOth” constant has been used as non-DDI candidate drug names. Moreover, “OtherNs (Drug)” is a function, which returns other generic or brand name of the Drug. Below shows the corresponding feature names alongside to the implemented rules:

- NeutralCandRule1-2: Two Boolean features are set true when the second drug name is a sample, a commercial brand or other common name of the first drug or both drugs belong to the same pharmacological class. For instance, in the sentence given in Fig 6, *Purinethol* is the brand name for *mercaptopurine*, and similarly *Imuran* for *azathioprine*:

The first Boolean (NeutralCandRule1) feature identifies textual patterns, where a “/” and a “(” separate the two drug names, and the second Boolean (NeutralCandRule2) feature detects textual patterns in which one of drug names contain another drug name or its synonyms. In both cases, the interaction status between the two recognized drug names is a valueless concept, hence a neutral candidate.

**Fig 7:**
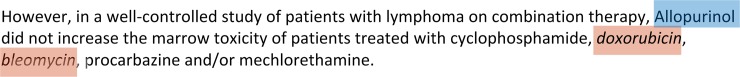
A sample of a sentence including a neutral and a distinguished DDI candidate.

- NeutralCandRule3-5: Three Boolean features are set true when an interaction between the two desired drugs with a third drug (or drugs) has been investigated; however, the interaction between the two drugs discussed has not been inspected. For instance, in the sentence presented in Fig 7, the interaction between *doxorubicin* and *bleomycin* (highlighted in red) has not been studied.

The first feature detects those drug candidates that have the same part of speech and grammatical roles (Object or Subject), and they are separated by “,” or “;” or an “additive transition” word. The second feature detects the case, where both drug names are samples of the same drug category (NeutralCandRule3). In this case, both drug names are mentioned after an introduction additive transition word, and they are also separated by “,” or an “additional additive transition” word.

The idea behind this category of features is that the interaction between two drug names, that have exactly the same “part of speech” and “grammatical role”, cannot be determined by the confident level more than zero. Therefore, the two drugs form a neutral candidate. Although these two features are the only patterns we could detect through analyzing textual language patterns, other similar features could possibly be extracted based on the similar “part of speech” and “grammatical roles” idea.

- NeutralCandRule6-10: Five Boolean features are defined for detecting those DDI candidates that are located in a clause (or sentence) with no additional information to the DDI, i.e. the lack of any investigation. We call these clauses non-informative clauses throughout this paper. Both dependent and independent clauses can be non-informative. Moreover, although non-informative clauses can have negation cue or do not have, the negated clauses have more neutral DDI candidates in comparison with non-negated clauses. For instance, in the following example, the sentence is non-informative, and the interaction between the drugs cannot be determined by the confident level greater than zero; consequently, the identified DDI candidates are neutral:
  ➢ “Pharmacokinetic interaction trials with cetirizine in adults were conducted by *pseudoephedrine*, *antipyrine*, *ketoconazole*, *erythromycin* and *azithromycin*.”

Taking neutral candidates into account is critical from another perspective, since not doing so may induce conflicts in the corpus later. For instance, in sentence presented in Fig 2, no investigation has actually been conducted into the possible interactions between *Propranolol* and *Ranitidine*, while such an interaction is considered as a negative DDI candidate in DrugDDI corpus. In this situation, the author did not make any remarks about the interaction between the two drugs, and it is possible that in the future, other researchers could find an interaction which would lead the corpus to face conflicts.

Ultimately, it is worth noting that the significant contribution of neutral candidates and features has been reconfirmed in our other research with other corpus [34]. Moreover, it is important to mention that the proposed neutral-related rules can be used with very slight change in other biomedical relation extraction tasks, especially symmetric relations such as protein-protein interaction. The first subcategory of neutral detection rules identify superficial patterns that can be applied to other biomedical domains. However, more patterns can be employed for identifying equivalent names of an entity in addition to the proposed patterns. The second subcategory of neutral features detects candidates that are located in non-informative sentences which may provide the background information or mention the objectives of the research which are common in biomedical articles. Finally, the third category detects candidates that occur more frequently in symmetric biomedical relations in which every the combination of entities can be a relation candidate.

### 3.4 Drug-drug interaction prediction

**Fig 8:**
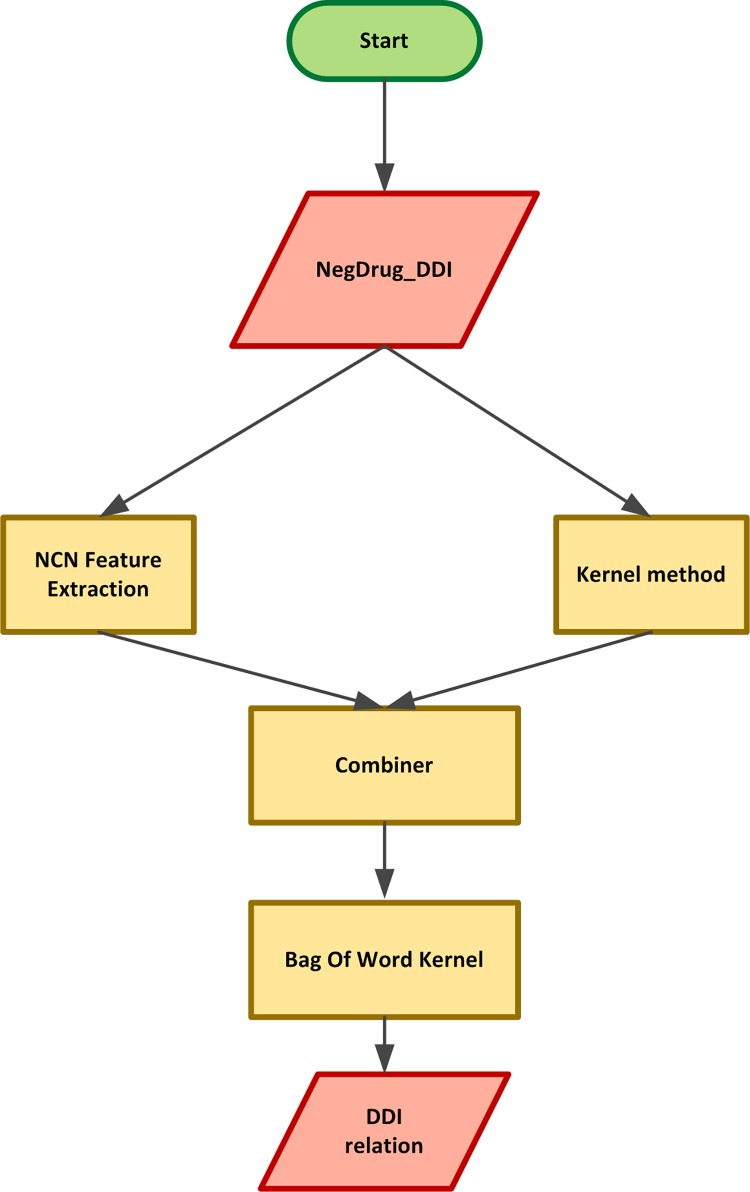
Basic components of the implemented framework.

Finally, the proposed method and different components of the system are discussed. The implemented framework is depicted in Fig 8. As the flowchart shows, the sentence, drug names, and negation scopes and cues extracted from the NegDDI corpus are employed as inputs for the three improved methods. Each of the three proposed methods consists of linear combination of the novel proposed features and the substructures of the kernel method (e.g. all tokens for global context kernel and subtrees for subtree kernel). During the experiments, the training parts of the DrugBank and MEDLINE of the corpus was utilized to train the classifiers, and the test part was used to test the system.

A support vector machine with *SMO* implementation [35] was applied, which performed likewise with *libSVM*, when the best setting of parameters was employed. *Weka* API was utilized as the implementation platform. The tokenization of the text features was executed without stemming process. Furthermore, in all of the above-mentioned methods, all the entities were considered as blind, replacing all the drug names in the generated features with two general terms, i.e. DrugName (for the two drugs whose interaction is being investigated) and *OtherDrugNames* (for the other drugs). Tokenization was carried out by the Stanford *BioNLPTokenizer* [36] which was adapted with pharmaceutical text, while the Stanford parser was used for constituent parsing. In addition, *TreeTagger* [37] was employed for Lemmatizing and POS tagging which were applied by the winning team in the DDI extraction challenge in 2011.

## Results

We first present our results of the comparison between the augmented method and the original method as well as the contribution of different features. Following that, the results of a statistical sign test for characterizing the significance of the obtained improvements will be presented. F-measure is selected as a single performance measure.

It is important to mention that the two datasets of the DDI corpus was utilized due to dealing with different types of texts and language styles [25]. DrugBank texts focus on the description of drugs and their interactions, while the MEDLINE text would not emphasis on DDIs. Table 2 demonstrates some of the basic statistics of the two used datasets.

**Table 2:** Basic statistics of the two utilized datasets of the DDI corpus.

	MEDLINE			DrugBank		
	Test	Train	Total	Test	Train	Total
**Documents**	33	142	175	158	572	730
**Sentences**	326	1301	2308	973	5675	6648
**Drug Names**	426	1836	2308	2512	12,929	15,441
**True DDI candidates**	95	232	327	884	3788	4672
**False DDI candidates**	356	1555	1911	4381	22,217	26,598
**Candidates with clause connectors**	126	478	604	2067	9215	11,282
**Number of Tokens**	14,358	61,525	75,883	244,658	1,163,072	1,407,730
**DDI Candidates with negation**	43	316	359	1367	4558	5925
**Total number of DDI candidates**	482	2033	1787	5265	31,432	36,697

### 4.1 Overall comparison of methods

The results of experiments that are similar to the SemEval DDI are presented in this section. In these results the training parts of the NegDDI-DrugBank and NegDDI-MEDLINE of the corpus was used to train the system, and the test parts were utilized to test the system.

Table 3 demonstrates the results for our improved global context (GC), subtree (ST), and local context (LC) kernel methods with NCT features in comparison with the standard methods. Four categories as well as the overall result are presented in that table as well: (i) those candidate sentences in the test part that have negation cue, but do not have any clause connectors, (ii) those with negation cue that have clause connectors, (iii) those without negation cue and with clause connector, and (iv) those without negation cue and clause connectors. The number of tested DDI candidates for each categories of sentence is presented in column four of the table.

**Table 3:** F1-measure results for Global Context (GC), SubTree (ST), and Local Context (LC) kernel methods with and without the NCT augmenting features.

	Category		Test Size	GC (%)			ST (%)			LC (%)		
				-	+NCT	↑	-	+NCT	↑	-	+NCT	↑
**DrugBank**	+Negation	-Connector	971	56.5	**62.2**	5.7	61.0	**68.5**	7.5	62.6	**65.2**	2.6
		+Connector	396	51.7	**58.4**	6.7	63.2	**63.4**	0.2	58.0	**63.1**	4.9
	-Negation	+Connector	1,005	62.3	**66.2**	3.9	58.6	**73.8**	15.4	64.8	**69.5**	4.8
		-Connector	2,893	64.8	**72.9**	8.1	36.3	**39.1**	2.8	63.9	**69.9**	5.8
	Total		5,265	61.7	**68.3**	6.5	47.1	**53.0**	5.9	63.4	**68.4**	4.9
**MEDLINE**	+Negation	-Connector	198	31.1	**39.2**	8.1	18.7	**22.7**	4	39.4	**50.6**	11.2
		+Connector	161	28.3	**38.2**	9.9	17.9	**23.1**	5.2	40.1	**50.1**	10
	-Negation	+Connector	443	34.6	**38.4**	3.8	18.9	**19.8**	0.9	44.2	**48.7**	4.5
		-Connector	1436	34.1	**40.6**	6.5	18.2	**18.4**	0.2	41.7	**44.8**	3.1
	Total		2238	33.9	**38.8**	5.9	18.4	**21.5**	3.1	42.3	**48.4**	6.1

The best result for the test part of the DrugBank part was achieved by the enhanced local context kernel method (LC+NCT), with 68.4% F-measure which is 2.7% more than the first system in DDI Extraction (2011) challenge (DrugBank part) with an F-measure of 65.7% that was implemented by the University of Trento, Italy.

In the global context and the local context kernel methods, the sentences without negation cues and clause connectors demonstrate the best improvement with an average of +8.1% (6.5% for MEDLINE part) increases in the F-measure. Moreover, in the subtree kernel method, the sentences without negation cues but with clause connectors indicate the best improvement with an average of +15.4% (+5.2% for MEDLINE part) increases in the F-measure.

We conclude that by using the proposed NCT features, not only the sentences with negation cues and clause connectors, but also the other sorts of sentences, including the sentences without negation cues and clause connectors benefit. As elaborated on in section 4.2, the main reason for this finding is neutral candidate features.

### 4.2 Contribution of each feature set

Table 4 shows that the proposed global context kernel with NCT features has the best performance in sentences that lack negation cues and clause connectors. The best improvement is gained by combining the neutral candidates and clause dependency features in the global context kernel, contributing 0.7% (for DrugBank part) more in the improvement process compared with the entire list of the invented features.

**Table 4:** F1-measure results for the global context kernel with combination of different feature sets: Negation scope and cue (N), Clause dependency (C), and neuTral candidate (T).

	Category		Global Context (%)							
			-	+N	+C	+T	+NC	+CT	+NT	+NCT
**DrugBank**	+Negation	-Connector	56.6	54.9	58.6	66.2	57.8	**67.2**	59.8	62.1
		+Connector	51.7	52.2	52.9	59.7	52.3	**59.8**	58.2	58.0
	-Negation	-Connector	64.7	64.8	64.8	71.8	64.8	71.9	71.9	**72.9**
		+Connector	62.3	62.3	65.3	65.3	63.7	**66.4**	65.7	65.9
	Total		61.7	61.3	62.9	68.6	62.4	**69.0**	67.5	68.3
**MEDLINE**	+Negation	-Connector	31.1	32.6	34.2	37.5	37.2	37.4	38.2	**39.2**
		+Connector	28.3	33.5	33.5	35.2	38.4	35.8	**39.4**	38.2
	-Negation	-Connector	34.6	38.7	35.4	35.4	36.1	34.8	37.5	**38.4**
		+Connector	34.1	38.2	36.7	37.2	35.4	36.4	39.5	**40.6**
	Total		33.9	36.6%	34.2	36.0	36.8	36.7	38.4	**38.8**

Our results concerning the proposed subtree (ST) kernel (Table 5) confirm that the dataset containing sentences without negation cues and with clause connectors has the best performance and the best rate of improvement (15.3% for DrugBank part). Although all feature sets improve the performance of the original subtree kernel, the best combination of features is neutral candidate and negation cue and scope features (Table 5), whose improvement is comparable to that of the entire list of features (15.3%). However, for those sentences containing negation cues, scopes, and connectors, no significant improvement was observed, possibly because the original subtree kernel had good performance for that type of sentences.

**Table 5:** F1-measure results for the subtree kernel with combination of different feature sets: Negation scope and cue (N), Clause dependency (C), and neuTral candidate (T).

	Category		SubTree (%)							
			-	+N	+C	+T	+NC	+CT	+NT	+NCT
**DrugBank**	+Negation	-Connector	60.9	59.2	59.9	66.9	68.9	59.9	**70.2**	68.5
		+Connector	63.2	63.1	62.6	63.2	62.7	63.2	63.1	**63.3**
	-Negation	-Connector	58.6	62.9	59.7	68.5	59.5	68.4	**73.9**	**73.9**
		+Connector	36.3	36.3	36.3	38.7	36.3	38.6	36.3	**39.1**
	Total		47.1	47.6	47.1	51.4	48.7	50.1	51.6	**53.0**
**MEDLINE**	+Negation	-Connector	18.7	19.9	20.2	20.8	19.8	22.6	**23.5**	22.7
		+Connector	17.9	19.4	19.6	19.6	17.3	18.7	20.7	**23.1**
	-Negation	-Connector	18.9	18.8	19.8	20.9	19.7	19.7	**20.7**	19.8
		+Connector	18.2	19.6	19.1	19.8	15.8	18.6	**20.6**	18.4
	Total		18.4	19.8	19.6	19.9	19.6	20.3	21.4	**21.5**

Finally, Table 6 indicates that the best combination of feature sets for the proposed local context (LC) kernel is neutral candidate with negation cue and scope features, producing slightly more improvement than the entire list of the invented features (68.5% for DrugBank and 48.3% for MEDLINE part). Furthermore, similar to the global context kernel, due to the consideration of tokens in the original version of the LC, negation scope and cue and clause dependency features generate some duplicated features which reduce the performance of the system. The high performance of neutral candidate features lifts up the overall performance of the feature set up to around +5%. Table 7 presents the f-measure results for test parts of the two used datasets as well as p-values which will be defined in the following section.

**Table 6:** F1-measure results for the local context kernel with combination of different feature sets: Negation scope and cue (N), Clause dependency (C), and neuTral candidate (T).

	Category		Local Context (%)							
			-	+N	+C	+T	+NC	+CT	+NT	+NCT
**DrugBank**	+Negation	-Connector	62.6	63.4	62.8	**66.0**	61.5	65.7	65.6	65.2
		+Connector	58.0	52.2	60.9	67.2	50.9	**67.8**	64.8	63.1
	-Negation	-Connector	64.8	65.9	64.9	66.2	65.7	66.9	68.9	**69.5**
		+Connector	63.9	65.3	63.9	69.6	64.2	**70.0**	69.9	69.9
	Total		63.4	64.1	63.7	68.1	63.0	**68.5**	**68.5**	68.4
**MEDLINE**	+Negation	-Connector	39.4	43.4	49.2	51.2	48.5	46.8	48.6	**50.6**
		+Connector	40.1	44.2	50.2	48.4	**53.8**	48.9	50.2	50.1
	-Negation	-Connector	44.2	37.2	42.5	42.6	45.9	**52.9**	44.7	48.7
		+Connector	41.7	48.4	43.2	49.8	46.1	47.3	**48.1**	44.8
	Total		42.3	43.5	45.7	46.5	47.7	**48.3**	47.9	48.2

**Table 7:** The f-score and calculated p-values by sign test for the test parts of the two datasets of the three improved and original methods.

	Method	-	+NCT (%)	M+	M-	*p*-value
**DrugBank**	**GC**	61.7	**68.3**	425	62	9.0e-53
	**ST**	47.1	**53**	395	65	3.6e-71
	**LC**	63.4	**68.4**	480	73	3.3e-64
**MEDLINE**	**GC**	33.9	**38.8**	143	35	2.0e-23
	**ST**	18.4	**21.5**	129	38	2.3e-32
	**LC**	42.3	**28.2**	153	34	3.4e-43

### 4.3 Sign test

To verify the significance of the proposed method, a sign test was conducted according to the approach of [22]: P = P r(X > Y); thus, the null hypothesis: H0: P = 0.50 was tested. For a given random pair of predictions by the original and the corresponding improved method (X_i_, Y_i_), the null hypothesis states that X_i_ and Y_i_ are equally prone to be larger than each other.

For calculating the sign test, we trained the systems with the training part of NegDDI-DrugBank and MEDLINE parts and tested them with the test part of the datasets. Table 7 depicts the p-values which state probabilities for accepting the null hypothesis.

In Table 7, column M+ shows the number of correct predictions by the improved method which have been incorrectly predicted by the corresponding original method and are considered a success. Column M- presents the number of correct predictions by the original method which has been incorrectly predicted by the corresponding improved method and is considered a failure. For instance, for the local context kernel, the calculated p-value is the chance of observing 480 successes in 553 trials.

Due to the p-value < 0.0001 in all the sign tests for all experiments, the null hypothesis is rejected and, as a result, all the improvements obtained are statistically significant.

### 4.4 Error analysis

In this subsection, two categories of errors are presented:

- **Inherent word ambiguities**. Although most of the clause connector features were successfully identified by the proposed system during the superficial features extraction process, few clause connectors features that have alternative speech parts in the sentence were identified with higher error rate. This happened because the extraction process of the superficial features only considers the structure of the texts rather than their semantics. For example, the connector”that” was the most problematic connector feature, due to the possibility of having different speech parts in the sentence, for example, being also a demonstrative pronoun. Thus, that was not used as a clause connector feature for simplicity.

Other clause connectors feature, similar to that, were considered or ignored, due to the common speech roles they take or do not, in scientific medical articles. For instance, the connector feature “when” was considered only as a connector, a common speech role in the mentioned articles, but ignored as an information question word. Consequently, in minor cases, the value of the feature was set to wrong value.

- **Parentheses**. Another source of inaccuracy in the proposed system as well as many of the text mining systems was parentheses. The error analyses of the system demonstrated higher rate of false positive in sentences with parenthesis. Several reasons contribute to the problem. For instance, parentheses are ignored in the negation annotation process, since the scope of annotation continues and cannot separate parenthesis from other parts of sentences. Consequently, the negation related feature was set to wrong value. For example, in some sentences in DrugDDI corpus, there is a clause or explanation containing the drug name that is placed inside parentheses such as the following sentence:
  ➢ Although specific drug or food interactions with *mifepristone* have not been studied, on the basis of the metabolism of these drugs by CYP 3A4, it is possible that *ketoconazole*, itraconazole, erythromycin, and grapefruit juice may inhibit its metabolism (increasing serum levels of mifepristone).

*Ketoconazole* and *mifepristone* are two drug names, which have been annotated as true interaction in the corpus. However, owing to the existence of parentheses, their interaction was not detected by the system. A sentence simplification algorithm could be useful to resolve the parentheses issue.

## Discussion and Future Works

In this paper, we studied a list of features including clause dependency features and some features for identifying neutral candidates as well as features extracted from negation cues and scopes. Our experiments indicate that the proposed features improve the performance of the relation extraction task combined with other kernel methods.

The obtained results show that the linguistically-oriented and scope-based negation annotation, which identifies negation cue and scope, does not generally yield sufficient information to decide upon negation confidently in the drug-drug interaction extraction. Therefore, one should regard other factors including identifying neutral candidates and clause dependencies. According to the results, neutral candidate feature set is the most useful among all three feature sets. In addition, better results are obtained from the combination of neutral candidate features with the other two feature sets.

Furthermore, as our analyses of the corpus show, sentences with negation cue have more clause connectors in comparison with sentences without negation cue; therefore, taking account of clause connectors and dependent clauses is important to solve the negation.

A stimulating question that has been partially answered in this work is whether all kernel methods benefit from the proposed features here. As our results of the subtree kernel for sentences with negation cues and clause connectors showed, it is possible that more advanced kernels using more informative features from different presentations of the sentence benefit less from the proposed features. In few experiments, the complete feature set did not yield the best results in comparison with other possible combinations of features. Thus, a suitable feature selection method can improve the results.

Moreover, in this work, some experiments for using a few basic simplification methods were carried out to overcome the complex sentences; for example by using the main clause as a separate feature, no significant improvement was achieved. However, a future work is trying a combination of simplification and pronoun resolution specified for drugs.

Another motivating future work is extension of the definition of the DDI relation and neutral candidate’s confidence level. The extension of the confidence level concept to a membership function for a fuzzy DDI relation instead of a crisp DDI relation will enable us to compare and combine extracted results from different sentences. Dissimilar results for a specific DDI candidate extracted from different sentences with different confidence levels can be compared and combined, which will contribute to identify different types of errors, including systematic or human ones. This can lead to boosting the overall performance of the system, which is not possible with a crisp DDI relation. Speculation and deduction cues including modal verbs of possibility, such as may and related adjective and adverbs, such as likely in addition to the proposed rule-based system to identify neutral candidates can be used to calculate the membership function, i.e. the confidence level.
